# Serum Folate Shows an Inverse Association with Blood Pressure in a Cohort of Chinese Women of Childbearing Age: A Cross-Sectional Study

**DOI:** 10.1371/journal.pone.0155801

**Published:** 2016-05-16

**Authors:** Minxue Shen, Hongzhuan Tan, Shujin Zhou, Ravi Retnakaran, Graeme N. Smith, Sandra T. Davidge, Jacquetta Trasler, Mark C. Walker, Shi Wu Wen

**Affiliations:** 1 Department of Epidemiology and Health Statistics, School of Public Health, Central South University, Changsha, Hunan, People’s Republic of China; 2 OMNI Research Group, Department of Obstetrics and Gynecology, Faculty of Medicine, University of Ottawa, Ottawa, Ontario, Canada; 3 Ottawa Hospital Research Institute, Clinical Epidemiology Program, Ottawa, Ontario, Canada; 4 Liuyang Maternal and Child Hospital, Department of maternal and child health care, Liuyang, Hunan, People’s Republic of China; 5 Division of Endocrinology, Department of Medicine, Mount Sinai Hospital and University of Toronto, Toronto, Ontario, Canada; 6 Department of Obstetrics and Gynecology, Queen’s University, Kingston, Ontario, Canada; 7 Women and Children’s Health Research Institute, University of Alberta, Edmonton, Alberta, Canada; 8 Department of Obstetrics and Gynecology, University of Alberta, Edmonton, Alberta, Canada; 9 Department of Physiology, University of Alberta, Edmonton, Alberta, Canada; 10 Department of Pediatrics, Faculty of Medicine, McGill University, Montreal, Quebec, Canada; 11 Department of Human Genetics, Research Institute of the McGill University Health Centre at The Montreal Children’s Hospital, Montreal, Quebec, Canada; 12 Department of Pharmacology and Therapeutics, Research Institute of the McGill University Health Centre at The Montreal Children’s Hospital, Montreal, Quebec, Canada; 13 School of Epidemiology, Public Health, and Preventive Medicine, Faculty of Medicine, University of Ottawa, Ottawa, Ontario, Canada; Xinhua Hospital, Shanghai Jiaotong University School of Medicine, CHINA

## Abstract

**Background:**

It has been reported that higher folate intake from food and supplementation is associated with decreased blood pressure (BP). The association between serum folate concentration and BP has been examined in few studies. We aim to examine the association between serum folate and BP levels in a cohort of young Chinese women.

**Methods:**

We used the baseline data from a pre-conception cohort of women of childbearing age in Liuyang, China, for this study. Demographic data were collected by structured interview. Serum folate concentration was measured by immunoassay, and homocysteine, blood glucose, triglyceride and total cholesterol were measured through standardized clinical procedures. Multiple linear regression and principal component regression model were applied in the analysis.

**Results:**

A total of 1,532 healthy normotensive non-pregnant women were included in the final analysis. The mean concentration of serum folate was 7.5 ± 5.4 nmol/L and 55% of the women presented with folate deficiency (< 6.8 nmol/L). Multiple linear regression and principal component regression showed that serum folate levels were inversely associated with systolic and diastolic BP, after adjusting for demographic, anthropometric, and biochemical factors.

**Conclusions:**

Serum folate is inversely associated with BP in non-pregnant women of childbearing age with high prevalence of folate deficiency.

## Introduction

Elevated blood pressure (BP) is a well-established risk factor for stroke and coronary heart disease [1], and the relationship between BP and cardiovascular mortality as well as overall mortality is continuous without a threshold [2]. In China, the incidence of hypertension significantly increased from 2.9 in 1990s to 5.3 per 100 person-years in 2000s [3], and there has been no slow-down in this trend [4].

Epidemiologic studies suggested that higher folate intake may be associated with decreased BP levels [5–7] as well as decreased incidence of hypertension [8, 9]. In these studies, folate intake was measured by food frequency questionnaire, which may lead to recall bias and misclassification of exposure. Little data is available on the association between blood folate concentrations and BP. Folate may have beneficial effects on BP by increasing nitric oxide synthesis and/or bioavailability in vascular endothelial cells [10–13], or by reducing plasma homocysteine [14, 15]. Animal studies have shown that oral folic acid supplementation improved endothelial function and decreased BP [16–18]. Altogether, these data suggest that folate intake may have an effect on decreasing BP. We therefore examined the association between serum folate and BP levels in non-pregnant Chinese women of childbearing age in a cross-sectional study.

## Materials and Methods

### Study design

We analyzed the baseline data of a cohort study of women of childbearing age in Liuyang county of Hunan province in China [19]. Participants who planned to have a baby within six months were being recruited from Liuyang Maternal and Infant Hospital at the time of marriage registration or premarital examination.

Participating women underwent a pre-gravid assessment which included: (1) interviewer- administered questionnaire (demographic characteristics and medical history); (2) physical examination (measurement of height, weight, and blood pressure); and (3) blood samples for measurement of serum folate, homocysteine, blood glucose, triglycerides, and total cholesterol.

### Measurements

The anthropometric measurements which included height and weight were standardized and performed by trained interviewers on a calibrated scale. Fasted blood samples were put on ice immediately after collection, transported to the laboratory within 30 minutes, and centrifuged at 4°C for 10 minutes at 3000 rpm. Tests were performed in central laboratory of Central South University using standard laboratory procedures.

Systolic blood pressure (SBP) and diastolic blood pressure (DBP) were measured by clinical staff at recruitment using automated non-invasive blood pressure monitors BpTRU, after the woman had rested for 10 min and were comfortably seated with the back supported. The upper arm was at heart level and supported, and an appropriate cuff was selected for the woman. BPTRU is an automated oscillometric non-invasive BP monitor that is reliable and reduces the “white-coat” effect [20]. Automatic mode was selected to obtain a series of six BP measurements, with a 2-minute interval between measurements. The clinical staff left the woman alone in the room after the first measurement. The first BP measurement was discarded, and the average BP value of the rest five measurements was obtained and recorded.

BP measurement and laboratory procedures were performed by different investigators, and all these measurements were blinded. Original records were double-entered and checked in order guarantee the quality of data.

### Statistical analyses

Serum folate was dichotomized according to the WHO’s definition of folate deficiency (< 6.8 nmol/L). Characteristics of the participants were described and compared across serum folate groups. Analyses of variance (for continuous variables) and chi-square tests (for categorical variables) were used to examine the differences.

Linear regression analyses were conducted to identify independent determinants of BP. Covariates included age, education, parity, body mass index (weight in kg / squared height in m^2^, or BMI), and biochemical profiles (serum folate, homocysteine, blood glucose, triglyceride, and total cholesterol). All variables were analyzed in crude models, and then included in multiple linear regression models. Multicollinearity diagnosis was obtained from the output of multiple linear regressions.

Principal component analysis was then performed because severe multicollinearity was detected in multiple linear regressions. Original data (BMI, serum folate, homocysteine, blood glucose, triglyceride, and total cholesterol) were standardized into z-scores, and scree plot was obtained from the principal component analysis. The principal components were extracted when eigenvalues exceeded 1. The component matrix was then obtained. Extracted components were included in multiple linear regression models as covariates in place of the original variables, adjusted for age, education, and parity, and a regression equation was obtained. Then, the principal components were transformed back to the scale of the actual covariates, using the selected principal components loadings (the eigenvectors corresponding to the selected principal components) to get the final regression estimator (with dimension equal to the total number of the original variables) for estimating the regression coefficients characterizing the original model. All analyses were performed in SAS version 9.2 (SAS Institute Inc., Cary, North Carolina). The significance level was 0.05 for all statistical tests.

### Ethics statement

The study protocol has been reviewed and approved by the institutional research ethics boards of Central South University (Changsha, China) and Ottawa Hospital Research Institute (Ottawa, Canada). The purpose, details and implication of the study were explained to participants. Written informed consents were obtained from all participants.

## Results

A total of 1,532 women recruited from April 2009 to February 2012 had completed all data on the baseline characteristics, including demographic, anthropometric, and biochemical measurements. Characteristics of the participants across serum folate groups are shown in Table 1. The mean age was 25.2 ± 3.1 (range 19–41), and mean BMI was 20.1 ± 2.4 kg/m^2^ (range 13.9–35.1). 845 women (55%) were folate deficient according to the WHO’s definition (< 6.8 nmol/L), and folic acid supplementation was nearly non-existent among them. Thirty women (2%) presented BP ≥ 140/90 mmHg. Women with folate deficiency had higher SBP and DBP levels.

**Table 1:** Characteristics of study population, by serum folate group.

	Overall	< 6.8 nmol/L	≥ 6.8 nmol/L	*P* ^a^
N	1522	845 (55.2%)	687 (44.8%)	
Serum folate (nmol/L)	7.52 ± 5.45	4.09 ± 1.75	11.74 ± 5.49	<0.01
Age (year)	25.21 ± 3.09	24.83 ± 2.77	25.69 ± 3.42	<0.01
Education (year)	10.05 ± 3.90	10.05 ± 3.96	10.05 ± 3.83	0.99
BMI (kg/m^2^)	20.11 ± 2.43	20.11 ± 2.46	20.12 ± 2.40	0.94
Homocysteine (μmol/L)	11.02 ± 4.10	11.36 ± 4.56	10.61 ± 3.42	<0.01
Blood glucose (mmol/L)	4.58 ± 1.25	4.47 ± 1.41	4.71 ± 1.02	<0.01
Triglyceride (mmol/L)	1.07 ± 0.71	1.10 ± 0.76	1.03 ± 0.64	0.05
Total cholesterol (mmol/L)	3.79 ± 1.14	3.69 ± 1.20	3.92 ± 1.06	<0.01
SBP (mmHg)	110.65 ± 11.48	112.28 ± 11.35	108.64 ± 12.87	<0.01
DBP (mmHg)	70.67 ± 8.72	71.50 ± 8.46	69.66 ± 8.93	<0.01
Parity				
Nulliparous	1312 (85.6%)	757 (89.6%)	555 (80.8%)	<0.01
Parous	220 (14.4%)	88 (10.4%)	132 (19.2%)	

BMI, body mass index; SBP, systolic blood pressure; DBP, diastolic blood pressure.

^a^ *P* values of analyses of variance for normal distributed variables, and chi-square tests for categorical variables.

Determinants of SBP and DBP are shown in Table 2. BMI and total cholesterol were positively associated with SBP and DBP, while age, parity, and serum folate were inversely associated with SBP and DBP. Regression diagnosis suggested severe multicollinearity in multiple regression models (eigenvalue < 0.01, condition number = 43.18).

**Table 2:** Determinants blood pressure in non-pregnant Chinese women.

	SBP (mmHg)			DBP (mmHg)		
	Crude (95%CI)	Adjusted (95%CI) ^a^	*P* ^b^	Crude (95%CI)	Adjusted (95%CI) ^a^	*P* ^b^
Serum folate (nmol/L)	–0.51 (–0.62, –0.39)	–0.45 (–0.57, –0.32)	<0.01	–0.28 (–0.36, –0.20)	–0.25 (–0.34, –0.16)	<0.01
Age	–0.48 (–0.67, –0.28)	–0.37 (–0.61, –0.12)	<0.01	–0.25 (–0.39, –0.11)	–0.23 (–0.41, –0.05)	0.01
Education	0.04 (–0.12, 0.19)	–0.06 (–0.27, 0.16)	0.59	0.12 (0.01, 0.24)	0.03 (–0.13, 0.18)	0.72
Parity	–4.79 (–6.53, –3.06)	–3.82 (–5.89, –1.75)	<0.01	–3.39 (–4.64, –2.15)	–2.82 (–4.31, –1.33)	<0.01
BMI (kg/m^2^)	0.46 (0.17, 0.76)	0.70 (0.39, 1.00)	<0.01	0.19 (–0.02, 0.40)	0.36 (0.14, 0.58)	<0.01
Homocysteine (μmol/L)	0.07 (–0.08, 0.23)	0.00 (–0.17, 0.17)	0.99	0.12 (0.01, 0.23)	0.07 (–0.05, 0.20)	0.24
Blood glucose (mmol/L)	0.14 (–0.38, 0.66)	0.08 (–0.51, 0.67)	0.79	–0.01 (–0.37, 0.36)	0.07 (–0.35, 0.50)	0.74
Triglyceride (mmol/L)	0.82 (–0.09, 1.73)	–0.32 (–0.14, 0.98)	0.58	–0.02 (–0.67, 0.64)	–0.92 (–1.75, –0.09)	0.03
Total cholesterol (mmol/L)	0.22 (–0.34, 0.78)	0.78 (0.15, 1.42)	0.02	0.42 (0.02, 0.82)	0.90 (0.45, 1.36)	<0.01

SBP, systolic blood pressure; DBP, diastolic blood pressure; CI, confidence interval; BMI, body mass index.

^a^ Adjusted for all other variables listed. Adjusted estimates can be interpreted in the following way: SBP decreased by 0.45 mmHg per unit (nmol/L) change in serum folate, after adjustment for all other variables.

^b^ *P* values of estimators in the adjusted model.

**Fig 1:**
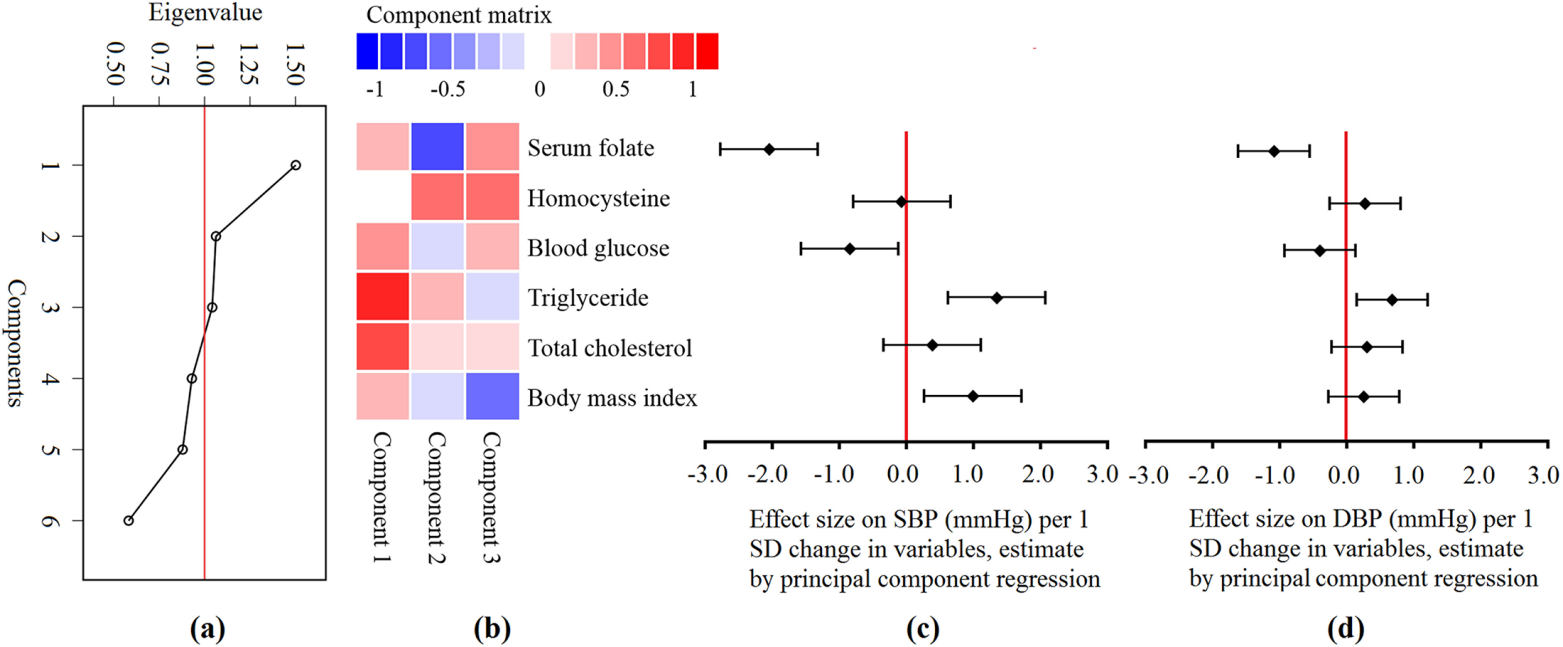
Estimates from principal component regression. (a) Scree plot shows the eigenvalue of each component. Components with an eigenvalue ≥1 were extracted. (b) Component matrix shows the correlation between z-scores of the original variables and principal components. Color signifies the direction and color depth quantifies the size, as indicated in the scale bar. (c) and (d) Effect size on SBP / DBP (mmHg) per one standard deviation (SD) change in variables, calculated according to the regression equations and component matrix (e.g. SBP and DBP decreased by 2 mmHg and 1 mmHg, respectively, per one SD increase in serum folate [nmol/L], after adjustment for all other variables).

Principal component regression was applied to deal with multicollinearity. Three principal components which explained 70% variation, were selected according to scree plot, as shown in Fig 1(a). Component matrix was calculated using standardized z-scores of all listed variables, as shown in Fig 1(b). Principal component regression was performed using these components instead of the original variables, adjusted by age, education, and parity. Multicollinearity was eliminated in the model. Then, regression coefficients of the original variables were calculated according to the component matrix and coefficients of principal components. Associations of SBP and DBP with determinants are shown in Fig 1(c) and 1(d). After adjusting for other variables (i.e. principal components), serum folate was inversely associated with SBP and DBP, while BMI and triglyceride were positively associated with SBP and DBP. One standard deviation increment in serum folate (nmol/L) resulted in approximately 2 mmHg decrease in SBP and 1 mmHg decrease in DBP. Parity was also associated with SBP (3.8 mmHg higher in nulliparous women) and DBP (2.8 mmHg higher in nulliparous women) (data not shown in Fig 1).

## Discussion

The main finding of this study was that serum folate level was inversely associated with BP levels in non-pregnant women of childbearing age. These associations were independent of adiposity, homocysteine, blood glucose, and lipids. Women with folate deficiency had higher BP levels. To our knowledge, this is the first study that reported an inverse association between serum folate and BP in non-pregnant women of childbearing age.

Few data are available on relationship of folate and BP, and the conclusion remains controversial. The HELENA study showed that red blood cell folate was positively associated with SBP in girls aged 12 to 17 in Europe, while serum folate was not associated with BP in either gender [21]. Other studies have examined the association between folic acid supplementation and BP or hypertension in various populations. A nutrient-wide association study using INTERMAP and NHANES data showed that dietary folate intake was inversely associated with DBP [7]. Similarly, a cross-sectional study in Japan showed that high intake of folic acid was associated with lower levels of BP among preschool children [5]. A double-blind placebo-controlled study in healthy postmenopausal Italian women showed that a three-week oral administration of 5-methyltetrahydrofolate reduced SBP by 5 mmHg [6]. The NHS II cohort study among US women found that higher folate intake was associated with a decreased risk of incident hypertension, especially among younger women [9]. The CARDIA cohort study in the US showed that higher folate intake in young adulthood was longitudinally associated with a lower incidence of hypertension later in life [8]. A meta analysis of randomized controlled trials showed that high-dose folic acid supplementation slightly lowered SBP in hypertensive patients [22].

The population of our study was quite different from other studies of the field: newly married non-pregnant women with a low prevalence of being overweight or obese (6%), but a high prevalence of folate deficiency (55%) [23]. Although the Chinese government provides free folic acid pills for women who are preparing to get pregnant, only four women in this cohort reported multiple vitamin or folic acid supplementation use. This is associated with limited health literacy in China: the 2012 National Health Literacy Survey showed that only half of the residents in Hunan province understood the benefits of folic acid supplementation [24]. In addition, folic acid fortification is not mandatory in China. Moreover, Liuyang is historically famous for preserved meat, dried and pickled long bean, stinky tofu (a form of fermented tofu that has a strong odor), chopped and salted chilli, and salted black bean [25]. Naturally occurring dietary folate will be lost in these pickled and smoked foods and over-cooked vegetables [26]. All of these factors may have contributed to the low blood folate level in this group of Chinese women.

The mechanism(s) by which folate is inversely associated with BP may involve oxidative injury and/or endothelial function. In a spontaneously hypertensive rat model, low dietary intake of folate resulted in increased SBP by 15 mmHg, and oxidative tissue damage in liver, heart and kidneys [16]. In Ang II-treated WT mice, folic acid treatment reduced high blood pressure, improved nitric oxide bioavailability and improved renal cortical blood flow [18]. In alcohol-treated Wistar rats, folic acid increased aldosterone clearance and prevented the increase in BP associated with chronic alcohol intake [17]. Folic acid can scavenge superoxide anions [10], enhance the enzymatic activity of endothelial nitric oxide synthase (eNOS) [11], and rescue or stabilize tetrahydrobiopterin (cofactor of eNOS) [12]; these processes result in increased synthesis and/or bioavailability of nitric oxide and vasodilation. However, the role of homocysteine as a mediator remains controversial since many studies have shown that folic acid improved endothelial function without any effects on homocysteine concentrations [27–29]. Although we observed a positive association between homocysteine and DBP, we failed to detect a significant association between serum folate and homocysteine.

There are limitations of the study. First, the causal inference is difficult to be made from the cross-sectional data, although dietary pattern is often considered as a long-term habit that is associated with socioeconomic determinants [30], so that low current serum folate may somehow reflect cumulative insufficient folate intake. A retrospective cohort study in European populations using one-year-recall food frequency questionaire showed that inadequate dietary folate intake was associated with subsequent low serum folate [31]. The HELENA study in European adolescents also suggested a positive association between serum folate and the self-reported intake of folate [32]. Second, genetic polymorphism (*MTHFR* C677T), which interferes with the absorption and metabolism of folate [26], was not determined. Women with *MTHFR* 677C→T variant usually have lower serum and red-blood-cell folate concentrations even when the folate intake is the same [33]. However, serum folate level is a comprehensive indicator of dietry folate intake, genetic polymorphism, and the interaction between these two factors [34]. Third, since the study population consisted of Chinese women of childbearing age, it remains to be determined whether our finding can be generalized to other populations.

Despite the limitations, our study has a number of strengths and implications. First, the study serves to address the gap in the literature with respect to the association between serum folate and BP. Second, all measurements were blinded: BP and serum folate concentration measurements were performed by different personnel. Neither of them had access to each other’s original records. Third, serum folate level was determined as a biomarker of internal exposure, hence recall bias in dietary survey as well as the interaction between dietary and genetic factors were avoided. Fourth, the statistical technique used in our study helped to solve the multicollinearity problem which was identified in our data. The issue of multicollinearity issue has been neglected in regression models in previous studies of the field.

In conclusion, serum folate concentration is inversely associated with BP in Chinese women of childbearing age, and this association is independent of adiposity, homocysteine, blood glucose, and lipid profile. Whether this association persists into pregnancy, and how it affects hypertensive disorders of pregnancy and cardiovascular disease in later life warrant investigation in future research.

## Supporting Information

S1 File Baseline data of the study. (XLSX)
